# Further confirmation of broadly conserved, highly immunogenic cross-clade HIV CTL epitopes for inclusion in the GAIA HIV vaccine

**DOI:** 10.1186/1742-4690-9-S2-P295

**Published:** 2012-09-13

**Authors:** AS De Groot, L Moise, O Koita, K Sangare, L Levitz, J Rozenhal, Z Koty, K Tounkara, M Ardito, CM Boyle, M Rochas, W Martin

**Affiliations:** 1 EpiVax, Inc, Providence, RI, USA; 2 University of Bamako, Bamako, Mali; 3 Institute of Immunology and Informatics University of Rhode Island, Providence, RI, USA; 4 GAIA Vaccine Foundation, Bamako, Mali

## Background

One of the biggest challenges for HIV vaccine design is identifying conserved HLA Class I- and Class II-restricted epitopes that would impart substantial fitness cost to the virus, thereby controlling or preventing infection. Here, we set out to develop an epitope-driven, DNA-prime/pseudoprotein-boost HIV vaccine (GAIA vaccine) composed of such epitopes.

## Methods

Analysis was performed in 2003 on 10,803 HIV-1 sequences, and again in 2009, on an expanded set of 43,822 sequences. These were searched for conserved 9-10-mer segments with the EpiMatrix suite of immunoinformatic algorithms. From the most highly conserved (>5% isolates) or top 1,000 scoring 9-mers, HLA Class I binding sequences and Class II immunogenic consensus sequences (ICS) were identified for vaccine design. Validation was performed in Thailand, the USA, and Mali via in vitro binding and IFN-γ ELISpot assays, using HIV-infected donor peripheral blood. HLA transgenic mice were immunized in DNA-prime/peptide-boost vaccine studies including these epitopes.

## Results

Epitopes selected as described are more broadly conserved than those selected for other epitope-based vaccines (>70%, compared to Epimmune’s 40%). Antigenicity of 98% of ICS epitopes and Class I epitopes (87% A2, 29% A3, 67% B7, 20% A24) was confirmed in HIV-infected subjects. Fifteen ICS peptides and 12 A2 peptides were confirmed in both Mali and the USA. Sixteen A3 peptides were confirmed weighting sequences for conservation and immunogenicity (as determined by EpiMatrix score), in vitro binding, and positive ELISpot responses. Epitope immunogenicity was validated in HLA transgenic mouse immunization studies.

## Conclusion

The GAIA vaccine approach is an effective means of triaging HIV epitope sequences to identify the most immunogenic and conserved epitope candidates across HLA allotypes. HLA transgenic mouse studies showed prototype DNA vaccines including these epitopes to be immunogenic, suggesting the epitope-rich GAIA vaccine would induce greater immunogenicity than other DNA/viral vector prime-boost vaccines in humans.

